# Addressing infectious challenges in pediatric cancer and hematopoietic cell transplantation: insights from the Umbrella Working Group

**DOI:** 10.1007/s00520-025-09337-5

**Published:** 2025-03-11

**Authors:** Luise Martin, Fabianne Carlesse, Caitlin W. Elgarten, Andreas H. Groll, Christa Koenig, Maria Kourti, Jessica E. Morgan, Zoi-Dorothea Pana, Loizos Petrikkos, Bob Phillips, Athanasios Tragiannidis, Eleana Vasileiadi, Roland A. Ammann, Eva Brack, L. Lee Dupuis, Daniel Ebrahimi-Fakhari, Alessio Mesini, Galina Solopova, Lillian Sung, Nadja Vissing, Thomas Lehrnbecher

**Affiliations:** 1 Charité – Universitätsmedizin Berlin, corporate member of Freie Universität Berlin and Humboldt-Universität zu Berlin, Medical Directorate, Berlin, Germany; 2 Department of Pediatric Respiratory Medicine, Immunology and Critical Care Medicine, Charité – Universitätsmedizin Berlin, corporate member of Freie Universität Berlin and Humboldt-Universität zu Berlin, Berlin, Germany; 3 Oncology Pediatric Institute - IOP-GRAACC-UNIFESP, São Paulo, Brazil; 4 Federal University of São Paulo, São Paulo, Brazil; 5 Division of Oncology, Children’S Hospital of Philadelphia, Philadelphia, USA; 6 Infectious Disease Research Program, Center for Bone Marrow Transplantation and Department of Pediatric Hematology/Oncology, University Children’S Hospital, Münster, Germany; 7 Pediatric Hematology/Oncology, Department of Pediatrics, Inselspital, Bern University Hospital, University of Bern, Bern, Switzerland; 8 Pediatric & Adolescent Hematology Oncology Unit, 2nd Pediatric Department, Faculty of Health Sciences, Aristotle University of Thessaloniki, AHEPA Hospital, Thessaloniki, Greece; 9 Candlelighters Supportive Care Research Centre, Centre for Reviews and Dissemination, University of York, York, UK; 10 Department of Paediatric Haematology and Oncology, Leeds Children’S Hospital, Leeds, UK; 11 Medical School, University of Nicosia (UNIC), Nicosia, Cyprus; 12 Department of Nursing, University of West Attica, Athens, Greece; 13 Pediatric Ambulatory Care - 1, Health Authority, Attica, NHS Greece; 14 Division of Infectious Disease, Department of Pediatrics, Children’S Hospital of Philadelphia, Philadelphia, PA USA; 15 Medical Faculty, University of Bern, Bern, Switzerland; 16 Statconsult Ammann, Burgdorf, Switzerland; 17 Research Insititute, the Hospital for Sick Children, Toronto, ON Canada; 18 Department of Pharmacy, The Hospital for Sick Children, Toronto, ON Canada; 19 Leslie Dan Faculty of Pharmacy, University of Toronto, Toronto, ON Canada; 20 Infectious Diseases Unit, IRCCS Istituto Giannina Gaslini, Genoa, Italy; 21 Infection Control Department, Dmitry Rogachev National Medical Research Center for Pediatric Oncology, Hematology and Immunology, Moscow, Russia; 22 Division of Haematology/Oncology, The Hospital for Sick Children, Toronto, ON Canada; 23 Department of Paediatrics and Adolescent Medicine, Copenhagen University Hospital – Rigshospitalet, Copenhagen, Denmark; 24 Department of Pediatrics, Division of Hematology, Oncology and Hemostaseology, Goethe University Frankfurt, Theodor-Stern Kai 7, 60590 Frankfurt/Main, Germany

Infectious complications remain one of the most challenging aspects of care for children and adolescents undergoing treatment for cancer or hematopoietic cell transplantation (HCT) and may be an independent risk factor for poor outcome [1]. Historically, supportive care strategies were largely determined by study groups and rarely extended across multiple countries or continents. To address this gap, the Umbrella Working Group was established in 2003 by international experts specializing in infections in pediatric oncology patients. Since then, this group has met regularly fostering collaboration and shaping future research directions and making significant contributions to the development of international pediatric-specific guidelines by many of the participants [2–6].

Over time, the Umbrella Working Group has evolved into a vital network of key opinion leaders in the management of infections among pediatric cancer patients. The most recent meeting was held on October 4–5, 2024, in Thessaloniki, Greece, bringing together experts from 12 countries. This report highlights the key topics discussed during the meeting, including both new proposals and updates on ongoing projects, thus offering valuable research priorities for scientific groups specialized in this field of pediatric oncology.

## Individual-patient-data meta-analysis on time-to-antibiotics (TTA) for febrile neutropenia

Timeliness of initiating antibiotic therapy is a well-established quality of care metric in febrile neutropenia. Based on limited data, the “golden hour” to start antibiotics has become the standard of care but may be unnecessary in a considerable proportion of patients. Recognizing the diversity of clinical settings, treatment protocols, and patient characteristics, the Umbrella Working Group collected data of more than 3500 febrile neutropenic episodes to perform a complex individual-patient-data meta-analysis (Christa Koenig, Switzerland). This ambitious joint venture aims to optimize time thresholds for initiating antibiotics in relation to patient survival and infection outcomes, factoring in variables like clinical condition at presentation, cancer type, depth and duration of neutropenia, and other individual risk factors.

## Antibiotic prophylaxis in acute leukemia

Another important discussion focused on the use of antibiotic prophylaxis during intensive treatment phases such as in acute myeloid leukemia. While this approach reduces infectious episodes, it remains controversial due to concerns of toxic adverse events and the development of resistance, both on an individual and institutional level. However, a randomized trial showed that antibacterial prophylaxis reduces exposure to broad-spectrum antibiotic therapy such as aminoglycosides and third or fourth-generation cephalosporins [7]. The outcome of breakthrough infections with levofloxacin-resistant pathogens is currently being analyzed (Eleana Vasileiadi, USA), and the results can be validated by another dataset already available to members of the panel. Another important question is the role of antibacterial prophylaxis in children undergoing induction treatment for acute lymphoblastic leukemia (ALL), a phase marked by profound immunosuppression through neutropenia and therapeutic corticosteroid use. Bob Phillips (UK) reported on the current status of a randomized study (CiproPAL Trial Identifier EudraCT number: 2021–000341-40), which assesses risks and benefits of ciprofloxacin prophylaxis in the induction phase of the ALLTogether clinical trial (Trial Identifier EudraCT number: 2018–001795-38).

## Feasibility of early discontinuation of empirical antibiotic therapy in HCT

Current pediatric-specific guidelines recommend with moderate evidence to consider discontinuation of empiric antibiotic therapy in low-risk patients without bone marrow recovery if patients have been clinically stable and afebrile for at least 24 h and blood cultures are negative at 48 h [6]. Evidence for this strategy in high-risk patients such as those with leukemia relapse or acute myeloid leukemia is less robust, but promising data were reported from a randomized study in adult high-risk patients including HCT recipients and from a retrospective analysis in children undergoing allogeneic HCT [8, 9]. Caitlin Elgarten (Philadelphia, USA) presented a proposal for early discontinuation of empiric antibiotics in pediatric patients undergoing HCT. This patient population is unique compared to those receiving conventional chemotherapy in that HCT recipients are hospitalized through engraftment, which offers closer monitoring than being at home, and no impact on quality of life by intravenous antibiotics. Clinical endpoint of this study would have to be adapted to the transplant setting and may be best addressed by a hierarchical composite outcome that considers infection resolution and relapse and antibiotic side effects. Another matter of discussion was the intervention specifics, e.g., using a step-down strategy to antibiotic prophylaxis vs. no antibiotics or decision-making based on targeting a specific neutrophil count. Implementing such trials could lead to more targeted antibiotic use, reducing adverse effects without compromising patient safety. Viral reactivations, including cytomegalovirus (CMV), Epstein-Barr virus (EBV), and human herpesviruses (HHV-6/7), were not a focus of discussion during the Thessaloniki meeting, although they are well-recognized complications in post-HCT patients. As these infections might interfere with the planned project, they should be included in the design of the study.

## Fever in the non-neutropenic patient with cancer

Fever in non-neutropenic children and young people with cancer represents approximately one-third to a half of febrile episodes in this patient population. Unfortunately, in contrast to febrile neutropenia, no evidence-based guidelines exist for this situation, and diagnostic and therapeutic approaches vary widely across the institutions. Although neutropenia is the most important single risk factor for severe infectious complications, non-neutropenic patients also have risk factors for infections such as the presence of a central venous line. Overall, several studies had described and validated risk factors for bloodstream infections in non-neutropenic febrile children with cancer [10, 11], but further work into risk stratification for this population and how this might be incorporated into clinical practice is needed. Through the Pediatric Oncology Group of Ontario (POGO) guidelines program, Paula Robinson (POGO), Priya Patel (POGO), Gabrielle Haeusler (Melbourne), and Jess Morgan (York) are considering whether to proceed with an evidence-based guideline, having performed a systematic review of published data. The advantages and disadvantages of drawing evidence from other groups (e.g., adult patients, or other children with central lines) were discussed. Despite these uncertainties, the panel recognized the high relevance of this topic and encouraged the development of an evidence-based guideline tailored specifically to the pediatric oncology population, utilizing the available data.

## Registries on rare fungal pathogens including resistance patterns

Compared to adults, children with cancer represent a small patient population, which limits the scope of most analyses and makes large-scale studies challenging. The Umbrella Working Group is therefore an ideal platform for standardizing and coordinating data collection globally [12]. In that respect, the panel agreed to focus on infections with rare yeast (Fabianne Carlesse, Sao Paulo, Brazil) and agents of mucormycosis (Loizos Petrikkos, Athens, Greece), but it was emphasized that collaboration with existing databases, such as the FungiScope [13, 14], is necessary. The primary objective is to enhance these registries by incorporating pediatric-specific data to enable more robust conclusions. Furthermore, solid evidence on the efficacy of newer antifungal agents, such as isavuconazole, in the treatment of invasive aspergillosis and mucormycosis is crucial for developing evidence-based guidelines (Athanasios Tragiannidis, Thessaloniki, Greece; Daniel Ebrahimi-Fakhari, Münster, Germany). The panel also emphasized the high priority of gathering and standardizing epidemiological data on emerging resistance among fungal pathogens, including candidemia in pediatric cancer patients (Zoi-Dorothea Pana, Cyprus). This analysis could support surveillance efforts and strengthen future antifungal stewardship (AFS) strategies in pediatric hematology-oncology. To this end, an online survey will be conducted within the Umbrella Working Group to assess AFS practices and prescribing behaviors (Maria Kourti and Athanasios Tragiannidis, Thessaloniki, Greece). However, challenges persist in comparing data due to variations in interpretation frameworks, such as those from the Clinical Laboratory Standards Institute (CLSI) in the USA and the European Committee on Antimicrobial Susceptibility Testing (EUCAST) in Europe.

## Conclusions and perspectives

The Thessaloniki meeting of the Umbrella Working Group highlighted current needs and challenged established practices in the prevention and treatment of infections in pediatric cancer patients, such as time-to-antibiotics, early antimicrobial discontinuation in the HCT setting, or the management of non-neutropenic febrile children. The panel vigorously discussed potential collaborative strategies to address these needs in a constructive atmosphere, fostering a productive exchange of ideas that will, hopefully, enhance the care of children and adolescents suffering from cancer. Progress on these topics will be discussed at the next meeting of the Umbrella Working Group meeting, scheduled for September 2025 in Berlin, Switzerland.

## Data Availability

No datasets were generated or analysed during the current study.
